# Immune-mediated competition benefits protective microbes over pathogens in a novel host species

**DOI:** 10.1038/s41437-022-00569-3

**Published:** 2022-11-09

**Authors:** Suzanne A. Ford, Georgia C. Drew, Kayla C. King

**Affiliations:** Department of Zoology, University of Oxford, 11a Mansfield Road, Oxford, OX1 3SZ UK

**Keywords:** Evolutionary ecology, Gene expression, Molecular evolution, Microbial ecology, Molecular ecology

## Abstract

Microbes that protect against infection inhabit hosts across the tree of life. It is unclear whether and how the host immune system may affect the formation of new protective symbioses. We investigated the transcriptomic response of *Caenorhabditis elegans* following novel interactions with a protective microbe (*Enterococcus faecalis*) able to defend against infection by pathogenic *Staphylococcus aureus*. We have previously shown that *E. faecalis* can directly limit pathogen growth within hosts. In this study, we show that colonisation by protective *E. faecalis* caused the differential expression of 1,557 genes in pathogen infected hosts, including the upregulation of immune genes such as lysozymes and C-type lectins. The most significantly upregulated host lysozyme gene, *lys-7,* impacted the competitive abilities of *E. faecalis* and *S. aureus* when knocked out. *E. faecalis* has an increased ability to resist lysozyme activity compared to *S. aureus*, suggesting that the protective microbe could gain a competitive advantage from this host response. Our finding that protective microbes can benefit from immune-mediated competition after introduction opens up new possibilities for biocontrol design and our understanding of symbiosis evolution. Crosstalk between the host immune response and microbe-mediated protection should favour the continued investment in host immunity and avoid the potentially risky evolution of host dependence.

## Introduction

Microbes that defend against infection by pathogens (along with parasites and parasitoids) colonise a large diversity of plant and animal hosts (Ford and King 2016; Kaltenpoth and Engl 2014; May and Nelson 2014; Parker et al. 2011). Protection can occur when symbiotic microbes suppress pathogen invasion by competing for resources/space or producing antimicrobial compounds (King 2019; Vorburger et al. 2013). Host-associated microbes can also suppress infection by modulating the host’s immune response to their benefit (Gerardo and Parker 2014). This immune-mediated competition can occur via the upregulation of host immune genes or components that play a role in disproportionately limiting the infection of pathogenic microbes over protective ones (Gerardo and Parker 2014; Mejía et al. 2014). This mechanism has been observed in animal-microbe symbioses. For example, the mosquito microbiome can modify basal immunity by upregulating immune genes conferring resistance to malaria parasites (Dong et al. 2009). Similarly, the commensal skin microbe, *Staphylococcus epidermis*, upregulates host immune responses that correlate with increased resistance to *Staphylococcus aureus* (Pastar et al. 2020). Immune-mediated competition has also been shown to structure diverse pathogen populations or communities by altering the competitive abilities of particular pathogen genotypes (Habets et al. 2012; Raberg et al. 2006; Ulrich and Schmid-Hempel 2012) or species (Bjørnstad and Harvill 2005; Lysenko et al. 2005; Margolis et al. 2010), respectively. The extent to which this mechanism of competition can operate at the origin of protective symbioses is unclear, particularly when protective microbes are invading a host species for the first time. Components of the host immune system—such as Toll- and NOD-like receptors, lysozymes and antimicrobial peptides—are known to regulate microbiota in *Hydra* (Bosch 2013), insects (Marra et al. 2021; Ryu et al. 2008), bobtail squid (Chen et al. 2017) and the mammalian gut (Mergaert 2018; Vaishnava et al. 2011), however there is a need for more information across diverse taxa and of the host control mechanisms specific to protective symbioses.

Interest in the mechanisms underpinning microbe-mediated protection has been surging because of its potential applicability in public health (O’Neill et al. 2018), species conservation (Trevelline et al. 2019) and agriculture (Singh et al. 2016). Although protective symbioses form naturally (Chrostek et al. 2017; Heath et al. 1999; Huigens et al. 2004; Jaenike et al. 2007), their artificial creation is being rapidly pursued for the biocontrol of infectious disease, either by introducing existing symbionts into new hosts (Bull and Turelli 2013) or by generating new symbionts via paratransgenesis (Magalhaes et al. 2019; Wang et al. 2017; Wilke and Marrelli 2015). For example, *Aedes aegypti* mosquitoes have been artificially infected with strains of the inherited symbiont *Wolbachia* that inhibit the transmission of arboviruses (Bian et al. 2013; Hoffmann et al. 2015; O’Neill et al. 2018). In the *Wolbachia* - mosquito system, the novel interaction upregulates host immune pathways and in turn, enhances antiviral protection (Rancès et al. 2012). It has been shown that such immune-upregulation can increase *Wolbachia* load (Pan et al. 2018), suggesting that immune-mediated competition could be occurring. Immune-mediated competition could facilitate the symbiont’s maintenance (Matthews et al. 2019) by allowing higher densities of symbiont to colonise. Higher densities of symbionts frequently correlate with greater protection (Drew and King 2022) and may feedback to promote maintenance of the symbiont. Over evolutionary time, the contribution of the host immune response may dictate the hosts vulnerability to pathogen infection should the protective symbiosis break down. Such break downs can be driven by imperfect transmission (Oliver et al. 2014), altered environment (Oliver et al. 2014; Raymann et al. 2017) or loss of protective traits (Alizon et al. 2013; Chrostek and Teixeira 2015; Ford and King 2016; Frank 1996; May and Nelson 2014). If protective microbes directly suppress infection, there is less need for hosts to defend themselves. This outcome could result in the divestment of costly host-based immune or damage response systems over evolutionary time (Ford and King 2016; Martinez et al. 2016; Metcalf and Koskella 2011; Parker et al. 2011). Conversely, host immune-mediated competition between protective and pathogenic microbes could maintain selection for host-based immunity (Mejía et al. 2014).

In this study, we investigated how a protective bacterial species (*Enterococcus faecalis*) could impact the transcriptomic response, and particularly the immune/defence responses, of novel *Caenorhabditis elegans* hosts to the pathogen, *Staphylococcus aureus*. These bacterivorous animals are genetically tractable with available immune knock-out mutants, and are thus useful for exploring the role of the immune response upon bacterial colonisation and infection. *Enterococcus faecalis* can be a pathobiont, ranging from being a gut commensal to opportunistic pathogen, and has been found to be protective in other animal hosts (Heikkilä and Saris 2003; Martín-Platero et al. 2006). It can colonise *C. elegans* guts through ingestion (Garsin et al. 2001). We have previously shown that *E. faecalis* can increase host survival during virulent infection by the opportunistic pathogen *Staphylococcus aureus* (King et al. 2016). Although we have demonstrated that this protective bacterium can quickly evolve to produce antimicrobial reactive oxygen species at levels to suppress *S. aureus* infection (King et al. 2016), other mechanisms are also at play. During co-colonisation, *E. faecalis* can inhibit *S. aureus* growth by stealing iron-binding siderophores that the pathogen releases into the environment (Ford et al. 2016). While neither *E. faecalis* nor *S. aureus* are known members of the native *C. elegans* microbiome, this tripartite system allows us to study how host and microbes interact at the inception of a novel symbiosis.

**Fig. 1:**
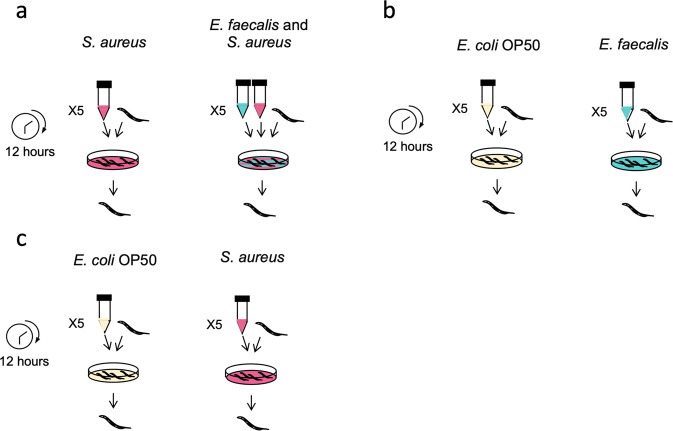
Experimental design for testing the effect of protective microbe *E. faecalis* and pathogen *S. aureus* on the transcriptional response of *C. elegans* hosts. Young adult worms were exposed to bacteria for 12 h to compare host transcriptional response between: (**a**) *S. aureus* in the absence vs. presence of *E. faecalis;* (**b**) *E. coli* OP50 (food control) vs. *E. faecalis;* and (**c**) *E. coli* OP50 (food control) vs. *S. aureus*. We included five replicate populations for each unique treatment (i.e. only a single set of five replicates were completed for *S. aureus* and *E. coli*, despite being shown twice in the figure). After 12 h exposure, the RNA from ~1000 worms per sample was sequenced.

We exposed populations of *C. elegans* to bacteria for 12 h to compare host transcriptional responses to: (a) *S. aureus* infection in either the absence or presence of protective *E. faecalis* (Fig. 1a); (b) the ‘food’ bacterium *Escherichia coli* OP50 or *E. faecalis* alone (Fig. 1b); and (c) the ‘food’ bacterium *E. coli* OP50 or *S. aureus* alone (Fig. 1c). These bacterial colonisers are thus horizontally acquired. Using RNA-sequencing, we found that the presence of *E. faecalis* in *S. aureus-*infected *C. elegans* caused the differential expression of 1557 genes in infected hosts, including the upregulation of immune genes that encode for lysozymes and C-type lectins. *E. faecalis* is reported to be much more robust to attack by host lysozymes, with a minimum inhibitory concentration (MIC) of >62.5 mg/ml lysozyme (Varahan et al. 2013), whilst *S. aureus* has lower reported MICs of 15 mg/ml (Cisani et al. 1982). We therefore hypothesised that lysozyme expression may facilitate immune-mediated competition between pathogen and protector. By using a host with one of the most highly upregulated lysozyme genes (*lys-7*) knocked-out, we show that lysozyme expression grants a competitive advantage to *E. faecalis* in pathogen-infected hosts. Expression of *lys-7* disproportionately suppresses the pathogen *S.aureus*, and as a result, facilitates enhanced protection by *E. faecalis*. This study shows that host immune responses can dictate the success of a protective microbe species when establishing in a novel competitive niche. Our work also highlights lysozymes as promising targets for manipulating microbe – microbe interactions within a host.

## Materials and methods

### Nematode host and bacteria

*Caenorhabditis elegans* is a nematode that ingests microbes for nutrients (Cabreiro and Gems 2013; Clark and Hodgkin 2014; Félix and Braendle 2010; Petersen et al. 2015) and is a well-established model for studying microbial colonisation and pathogenesis (Gravato-Nobre and Hodgkin 2005; Hope 1999; Montalvo-Katz et al. 2013; Peleg et al. 2008; Portal-Celhay and Blaser 2012). We used the simultaneous hermaphroditic N2 wild-type *C. elegans* strain from the Caenorhabditis Genetics Centre (CGC, University of Minnesota) along with a knock-out mutant for the *lys-7* gene that is outcrossed into the N2 genetic background (strain CB6738, CGC). We generated genetically homogenous lines by selfing a single hermaphrodite for five generations and froze them in 50% M9 solution and 50% liquid freezing solution in cryotubes at −80 °C (Hope 1999). We regularly resurrected populations throughout experimentation to prevent the accumulation of *de novo* mutations in host populations. Worms were maintained at 20 °C on 9 cm nematode growth medium (NGM) plates seeded with *Escherichia coli* OP50. *E. coli* OP50 is grown at 30 °C shaking at 200 rpm overnight in lysogeny broth (LB) and 100 µl of this is spread onto NGM plates and incubated overnight at 30 °C (Hope 1999). To ensure clean stocks and to synchronise the life stages of populations for experimentation, we treated worms with bleach (NaClO) and sodium hydroxide (NaOH) solution which kills everything except unhatched eggs (Hope 1999).

We used *Staphylococcus aureus* strain MSSA476 (GenBank: BX571857.1), an invasive community-acquired methicillin-susceptible isolate, as the pathogen in our system. *C. elegans* likely encounter *Staphylococcus* species in their natural habitat (Montalvo-Katz et al. 2013; Rossouw and Korsten 2017), however specific interactions with *S. aureus* here are considered novel as this species has not been recorded interacting with natural populations of *C. elegans*. As the protective microbe, we used *Enterococcus faecalis* OG1RF (GenBank: CP002621.1), an isolate from the human digestive tract. *E. faecalis* is naturally protective in a variety of animals (Heikkilä and Saris 2003; Martín-Platero et al. 2006) but is not a known member of the *C. elegans* native microbiome. Both *E. faecalis* and *S. aureus* are horizontally acquired by *C. elegans* through ingestion (Garsin et al. 2001; Sifri et al. 2003). We grew each species from a single colony overnight in 6 ml Todd Hewitt Broth (THB) shaking at 200 rpm at 30 °C. Bacteria were frozen in a 1:1 ratio of sample to 50% glycerol solution in cryotubes at −80 °C.

### Experimental set-up and RNA extraction

We exposed populations of young adult worms to bacteria for 12 h to compare host transcriptional responses to: (a) *S. aureus* in the absence vs. presence of *E. faecalis* (Fig. 1a); (b) *E. coli* OP50 (food control) vs. *E. faecalis* (Fig. 1b); and (c) food control vs. *S. aureus* (Fig. 1c). Five replicate populations were used for each unique treatment. We collected sterile and age-synchronised eggs using the bleach-sodium hydroxide solution. We kept these eggs in M9 buffer without food, shaking for ~8 h at 88 rpm and 20 °C to arrest development at L1. We transferred ~5000 L1 worms per replicate population to 9 cm NGM plates seeded with *E. coli* and placed at 20 °C to grow for 2 days. We chose this density to avoid overcrowding and starvation. Bacteria were grown from frozen culture overnight in 6 ml culture (THB for *E. faecalis* and *S. aureus*, LB for *E. coli* OP50) in a shaking incubator at 30 °C at 200 rpm and standardised their optical density to OD600 of 1 which corresponds to ~1 × 10^9^ cells/mL. We spread 120 μl culture per species onto 9 cm Tryptic Soy Broth (TSB) agar plates and incubated them overnight at 30 °C. Where worms were to be exposed to both *E. faecalis* and *S. aureus*, we mixed 120 μl of each culture on the same TSB plate. We removed young adult stage worms from the NGM plates, washed them in 50 ml M9 buffer five times and placed ~2000 young adults on the exposure plates at 25 °C for 12 h. At no time did the worms ever run out of bacteria to eat.

After 12 h of exposure, we washed worms off each plate using M9 buffer within 10 min in an order determined by a random number generator. We chose 12 h of exposure to avoid host mortality but provide sufficient time for *C. elegans* to respond to bacterial exposure and infection. We washed the worms in 10 ml M9 buffer five times and put ~1000 worms in 50 μl into Eppendorf tubes containing 1 ml Trizol and vortexed for 20 s. We then freeze-thawed the samples of worms three times using dry ice and a heat block (40 °C) to break the worm cuticle and stored at −80 °C. We extracted RNA using Zymo spin columns, following the manufacturer’s instructions with on-column DNA digestion using DNase I.

### RNA sequencing

We checked the quality of the RNA using an Agilent 2100 Bioanalyzer with the Eukaryote total RNA pico chip. We quantified the resulting RNA using the Qubit^®^ Fluorometer (Invitrogen) and all samples were diluted to the same final concentration. This was done to ensure that each sample is represented evenly for accurate transcript quantification. The Oxford Genomics Centre then performed library preparation and sequencing. The polyA signal was used to select the mRNA fraction from the RNA to capture just *C. elegans* and not the bacteria from the sample. RNA was then converted to cDNA. Second strand cDNA synthesis incorporated dUTP and the cDNA was then end-repaired, A-tailed and adapter-ligated. Prior to amplification, samples underwent uridine digestion. The prepared libraries were size selected, multiplexed and checked for quality before paired-end sequencing using NovaSeq6000 with 150 bp paired-end reads. On average, each replicate sample generated 22,588,105 reads, of which 82.55% were assigned to *C. elegans* and 4.46% to bacteria.

SortMeRNA (Kopylova et al. 2012) was used for further filtering of ribosomal RNA prior to alignment, on average each sample had 1.26% reads mapping to small, and 4.65% to large, subunit eukaryote rRNA sequences. Full detail on the number of reads associated with each run are shown in SI File 5.

We checked raw reads for quality using FastQC (0.11.5). Current (release 96) GTF and cDNA FASTA files were downloaded from the ensemble database for *C. elegans* (WBcel235 version of the *C. elegans* reference genome). We created a transcript index using Kallisto and the *C. elegans* WBcel235 cDNA FASTA file. We then performed pseudoalignment using Kallisto with 100 bootstraps and calculated transcript abundances. Kallisto pseudoalignment is a fast and accurate way of quantifying transcript abundance since it matches sequences to existing transcripts from a reference library, rather than performing a full alignment to the genome. This means that it tells you ‘what’ transcript a sequence is compatible with, but not ‘where’. These data are sufficient for transcript abundance quantification. We then used the R package Sleuth to perform statistical analyses (see ‘statistical analysis’).

### Host mortality

We tested whether the *lys-7* gene played a role in host mortality in response to *S. aureus* and *E. faecalis* colonisation and co-colonisation. We grew age-synchronised eggs to young adult stage on 9 cm NGM plates seeded with a lawn of *E. coli* OP50. Using the same protocols as describe above, we grew *E. coli* OP50*, S. aureus* and *E. faecalis* in vitro overnight, standardised the cultures to OD600 of 1, and made the exposure plates. We washed ~250 young adults of either CB6738 (*lys-7* knock-out) or N2 wild-type in 50 ml M9 five times before exposing them at 25 °C for 24 h. The proportion of dead worms were then counted as a measure of host mortality.

### Bacterial colonisation

We measured the co-colonisation ability of *E. faecalis* and *S. aureus* in wild-type worms and *lys-7* gene knockout worms. We exposed CB6738 (*lys-7* knock-out) and N2 wild-type worms to both *S. aureus* and *E. faecalis* together following the same protocol as above (see ‘Host mortality’). After 24 h of exposure to the bacteria, we collected 7–10 live worms per exposure plate and washed them in 5 ml of M9 five times under the microscope. To release the colonising bacteria, we placed the worms in 2 ml screwcap tubes with 50 μl M9 and 1.5 mm Zirconium beads (Benchmark Scientific) and broke the cuticle by shaking the tubes at 320 rpm for 45 s. We plated serial dilutions onto Mannitol Salt Agar to isolate *S. aureus* and TSB with 100 μg/ml rifampicin (Sigma-Aldrich) to isolate *E. faecalis*. We incubated the plates at 30 °C overnight before counting the number of colony-forming units (CFUs) per host.

### Statistical analysis

We performed differential expression analysis on the transcript abundance outputs from Kallisto using Sleuth in R v 3.2.0 (http://www.r-project.org/) and following the same format as previously published work on this system (Ford and King 2021). Sleuth uses generalised linear models to identify coefficients of the strength of expression. To identify differentially expressed transcripts between two experimental treatments, Sleuth compares the full model to a reduced model that assumes abundances are the same across the treatments (Pimentel et al. 2017), we then ran likelihood ratio tests of fitted models. This process was completed for three comparisons (*S. aureus* in the absence vs. presence of *E. faecalis*, OP50 vs. *E. faecalis* and OP50 vs. *S. aureus*) with treatment modelled as the dependent variable. SI files 1, 3 and 4 show DEG results from sleuth models and LRTs. The significance of treatment was determined by a *q* value of <0.05 (*p* value adjusted by means of the Benjamini-Hochberg false discovery date, FDR, correction for multiple comparisons). We performed a gene ontology (GO) term enrichment analysis on the significant differentially expressed genes using the g:Profiler online tool with the Benjamini-Hochberg FDR correction for multiple comparisons (Raudvere et al. 2019).

We used parametric tests for all data which met the required assumptions. We used the Shapiro test to detect whether data was normally distributed and *F*-tests to compare the variances of two samples from normal populations. We compared bacterial CFUs per host using two-sample *t*-tests. We used a binomial GLM to compare host mortality among host strains colonised by *E. faecalis* and *E. coli* OP50. Models were checked for overdispersion via the ratio of residual deviance to residual degrees of freedom. To account for overdispersion, we used quasibinomial GLMs to compare mortality among co-colonised host strains and host strains colonised by *S. aureus* singularly. In all mortality models the proportion of dead worms was modelled as a function of host strain (*lys-7* mutant or wild-type N2). We assessed the fit of GLMs by checking deviance values and diagnostic plots of residuals and quantile-quantiles. Hosmer-Lemershow goodness of fit tests were used to check there were no significant differences between the fitted model and the observed data. We corrected p-values where multiple comparisons were made from the same dataset using the FDR (Benjamini and Hochberg) method.

## Results

### Gut colonisation by *E. faecalis* alters host response to virulent infection

**Fig. 2:**
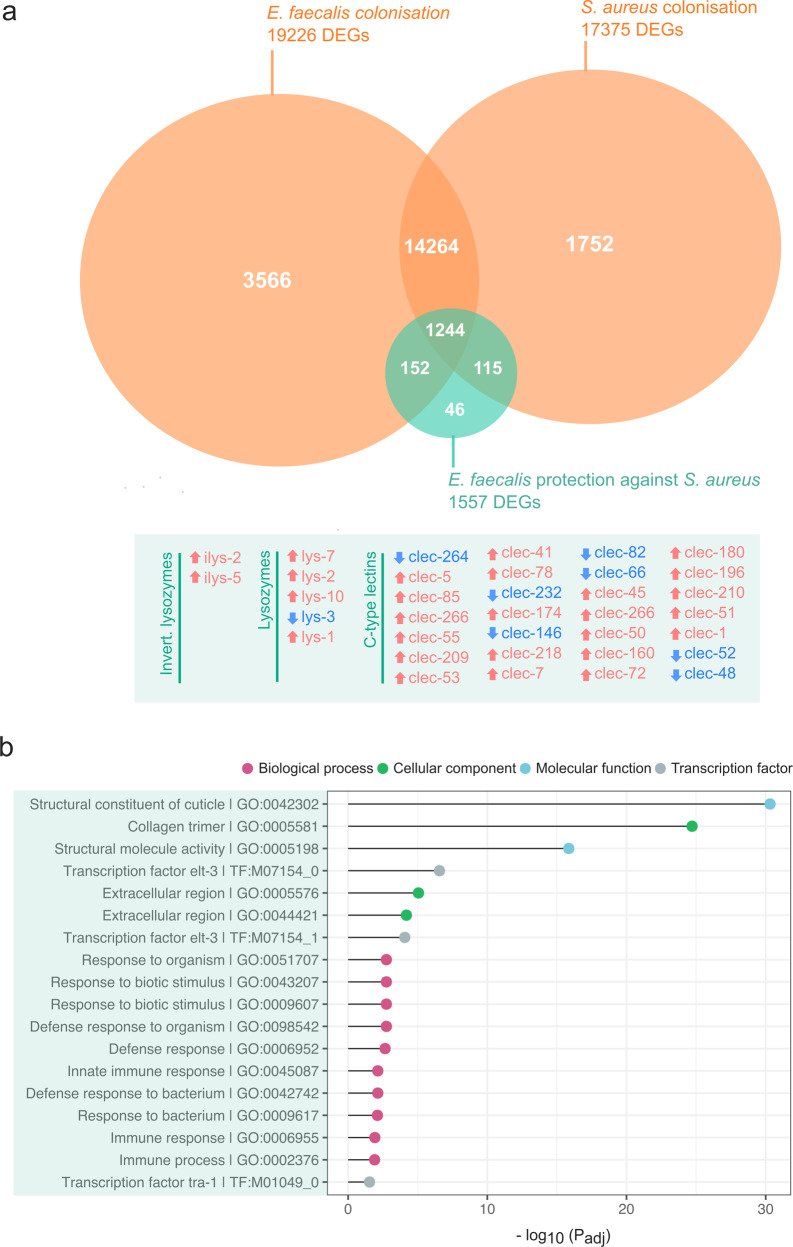
Differential host gene expression and gene ontology analysis under *E. faecalis*-mediated protection. **a** Differentially expressed genes (DEGs) of host *C. elegans* under single colonisation (orange) by *E. faecalis* and *S. aureus*, relative to co-colonisation (green) whereby *E. faecalis* protects against the *S. aureus* pathogen. The 46 immune gene families differentially regulated by *E. faecalis*-mediated protection are detailed in the green box, blue arrows indicate downregulation and red upregulation. See supplementary files 1, 3, 4 and 7 for further detail on DEGs across treatments. **b** Gene ontology (GO) terms significantly enriched in the list of *C. elegans* genes differentially regulated under co-colonisation and *Enterococcus faecalis-*mediated protection. Enrichment analysis performed using g:Profiler with Benjamini-Hochberg FDR correction for multiple comparisons.

We used RNA-sequencing to assess the transcriptional changes in *C. elegans* hosts during infection by virulent *S. aureus*, in the presence (co-colonisation) vs. absence (single colonisation) of *E. faecalis* (Fig. 1a). We found that the presence of *E. faecalis* drove significant differential expression of 1557 nematode genes compared with *E. faecalis’* absence (Fig. 2, Supplementary File 1). Of these genes, 521 were downregulated, whilst 1036 were upregulated. We performed a GO-term enrichment analysis on this list of genes and identified significantly enriched GO-terms in biological processes including “defence response to bacterium” (GO:0042742, FDR-adjusted *P* = 6.12E−09, Supplementary File 2) and “innate immune response” (GO:0045087, FDR-adjusted *P* = 4.09E−12, Supplementary File 2). We also found significantly enriched GO-terms in molecular functions including ‘structural constituent of cuticle’ (FDR-adjusted *P* = 1.02E−37, Supplementary File 2).

We found that the enriched GO-terms (Fig. 2b). relating to defence and the immune response were linked to numerous differentially expressed lysozyme-encoding genes (Fig. 2a). Lysozymes are important in the host defence response to bacteria as these enzymes break down bacterial cell walls (Ragland and Criss 2017). We found that the presence of *E. faecalis* in infected hosts was associated with the upregulation of the invertebrate lysozyme genes, *ilys-2* and *ilys-5* compared to when hosts were only infected by *S. aureus* (*ilys-2* beta = 1.171, *P* = 6.28E−06; *ilys-5* beta = 0.85, *P* = 0.00055). We also found a significant upregulation in the lysozyme genes, *lys-1* (beta = 0.77, *P* = 0.00028), *lys-2* (beta = 0.78, *P* = 3.01E−05), *lys-7* (beta = 2.12, *P* = 1.15E−07) and *lys-10* (beta = 3.46, *P* = 4.36E−05). Only *lys-3* was significantly downregulated (beta = −1.2, *P* = 0.00025). The most significantly upregulated lysozyme gene was *lys-7*. By measuring how *E. faecalis* alone alters host transcription compared to an *E. coli* food control (Fig. 1b, Supplementary File 3), we found that of these lysozyme genes, *E. faecalis* only upregulated *lys-1* (beta = 0.017, *P* = 0.0395). This result suggests that *E. faecalis* colonisation alone is sufficient to upregulate *lys-1* in nematodes, whilst *lys-2, lys-7* and *lys-10* also require pathogen presence to be upregulated. By measuring how *S. aureus* infection alone alters host transcription compared to an *E. coli* food control (Fig. 1c, Supplementary File 4), we found that of these lysozyme genes, *S. aureus* only caused the downregulation of *lys-1* (Y22F5A.4.1, beta = −1.0, *P* = 0.000009; Y22F5A.4.2, beta = 0.033, *P* = −0.92) and left the others unaffected. This result demonstrates that the presence of *E. faecalis* and *S. aureus* appears to impact *lys-1* in opposing directions whilst *lys-2, lys-7* and *lys-10* in nematodes require the co-colonisation of *E. faecalis* and *S. aureus* for upregulation.

In addition to lysozymes, the enriched GO-terms relating to defence and the immune response were connected with 28 differentially expressed C-type lectin (*clec*) transcripts (Fig. 2). Colonisation of *E. faecalis* during pathogen infection was associated with the upregulation of 20 *clec* genes and the downregulation of a further seven *clec* genes compared to when hosts were infected by only *S. aureus* (Fig. 1a, Fig. 2). These genes are carbohydrate-binding proteins that play a host defensive role against gram-positive bacteria (Pees et al. 2017). By comparing the impact of *E. faecalis* alone on host gene expression to gene expression in *E. coli* control hosts (Fig. 1b, Supplementary File 3), we found that colonisation by this bacterium was not sufficient to explain these results. Some of these nematode *clec* genes were not differentially expressed by *E. faecalis* alone, whilst *clec-41* (downregulated) and *clec-146* (Y48E1B.9b.1, upregulated) were regulated in the opposite direction to that seen during pathogen infection. It therefore appears that the expression profile of the 28 *clec* transcripts cannot be caused by *E. faecalis* colonisation alone, but is likely shaped by *E. faecalis* and *S. aureus* co-colonisation (Supplementary Files 3 and 4).

The enriched GO-terms relating to structural constituents of the cuticle were linked to many differentially expressed cuticular collagen genes (Supplementary File 2). We found that colonisation by both *E. faecalis* and *S. aureus* was associated with the upregulation of 44 *col* and 8 *dpy* genes compared to when hosts were only infected by *S. aureus* (Fig. 1a, Supplementary File 1). Both *col* and *dpy* genes are upregulated in response to pathogens which produce extracellular proteins that degrade collagen and weaken the host hypodermis (Sellegounder et al. 2019). This process facilitates pathogen invasion of the host and can be important for resistance to multiple pathogens, including *S. aureus* MSSA476 (Sellegounder et al. 2019). By comparing the transcriptional response of *C. elegans* in response to colonisation by *E. faecalis* alone versus a food control (Fig. 1b, Supplementary File 3), we discovered that *E. faecalis* appears to be sufficient to upregulate some *col* genes (*col-17, col-88, col-97* and *col-167*, Supplementary File 3). Of these, we found that *S. aureus* alone does not cause the upregulation of *col-17* or *col-88*, but does upregulate *col-97* and *col-167* compared to when nematodes are exposed to a food control (Supplementary File 4). This result shows that two upregulated cuticular collagen genes (*col-17* and *col-*88) are upregulated solely by *E. faecalis* colonisation, whilst another two can be upregulated by either bacterium (*col-97* and *col-167)*. Upregulation of the remaining genes is likely to require the coinfection of *E. faecalis* and *S. aureus*. However, we do not exclude the possibility that the host transcriptomic response observed under coinfection may be affected by an overall higher dosage of bacteria (combined dose of *E. faecalis* and *S. aureus*).

### Lysozyme expression determines outcome of competition between protective microbes and pathogens

Lysozymes are a well-known conserved antimicrobial defence (Ragland and Criss 2017). Because *E. faecalis* is more resistant to lysozyme activity than *S. aureus* (Cisani et al. 1982; Varahan et al. 2013), we hypothesised that lysozyme expression would disproportionately impact their competitive ability. Lysozymes could thereby contribute to host immune-mediated competition between the bacterial species and give protective *E. faecalis* an advantage. We focused on the impact of the most significantly differentially expressed lysozyme gene, *lys-7*, on bacterial colonisation and protection.

**Fig. 3:**
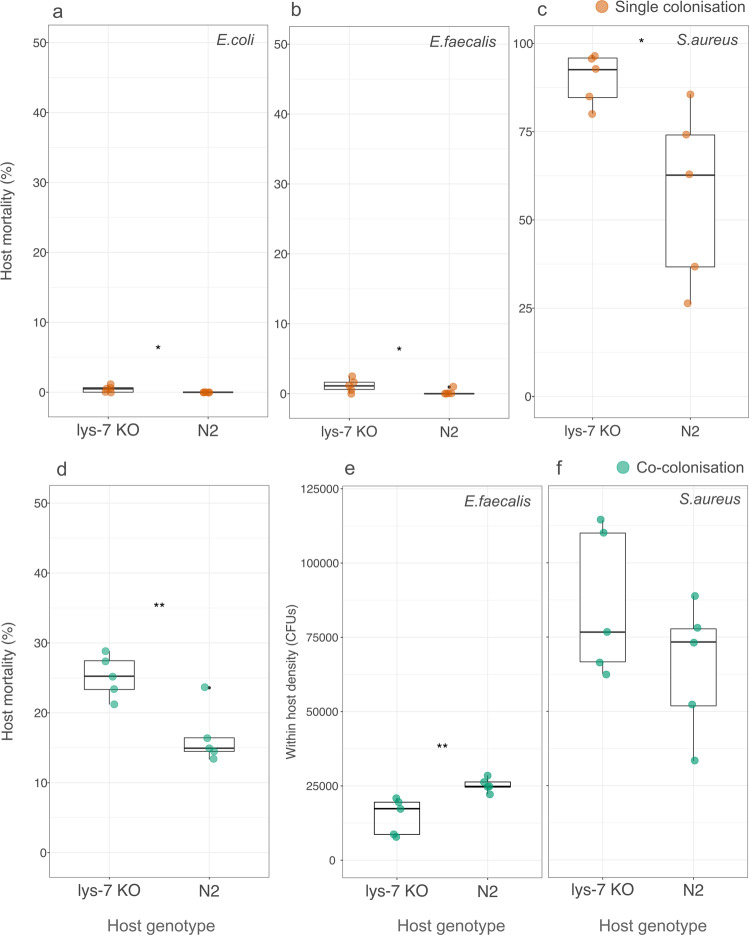
The effect of the *lys-7* gene on *C. elegans* mortality and bacterial colonisation. Percentage mortality of the *lys-7* knock-out mutant and wild-type N2 *C.elegans* host after single colonisation (orange) by food control *E. coli* (**a**), protective *E. faecalis* (**b**) and pathogenic *S. aureus* (**c**) and after co-colonisation (green) whereby E. faecalis protects against pathogenic *S. aureus* (**d**). Within-host bacterial density in colony-forming units (CFUs) of *E. faecalis* (**e**), and *S. aureus* (**f**) after 24 h of co-colonisation. Each treatment was replicated five independent times and 7–10 worms were collected per replicate for CFU quantification. *P < 0.05, **P < 0.01, ***P < 0.001.

We first tested whether knocking out *lys-7* increased host mortality to *E. faecalis, S. aureus* and a *E.coli* OP50 (food-only) control. We expected *lys-7* knock-out hosts to have increased mortality compared to wild-type N2 hosts and that this mortality would be much higher with the pathogen, *S. aureus*, than *E. faecalis*. As expected, knocking out *lys-7* increased host mortality across all bacterial exposures, however this increase was only small in the *E. faecalis* treatment and *E.coli* food control as these bacteria have very low virulence in this system (Fig. 3. *E. coli* contro*l*: Binomial GLM, df = 1, FDR-corrected *P* = 0.04; *E. faecalis:* Binomial GLM, df = 1, FDR-corrected *P* = 0.04; *S. aureus:* Quasibinomial GLM, *F* = 11.2, df = 1, FDR-corrected *P* = 0.03). In the *lys-7* knock-out, host mortality after 24 h exposure to food or *E. faecalis* remained very small (from 0% in N2 to 0.4%, and from 0.2% in N2 to 1.2%, respectively), but mortality caused by *S. aureus* infection increased greatly (from 57.6% in N2 to 90.3% in *lys-7* knockout nematodes). We then tested whether knocking out *lys-7* increased host mortality when co-colonised by *S. aureus* and *E. faecalis*. We found that host mortality was higher in *lys-7* knock-out hosts than in the wild-type (Fig. 3d. Quasibinomial GLM. host strain: *F* = 14.7, df = 1, *P* = 0.05). The protective effect of *E. faecalis* was maintained in *lys-7* knock-outs, with mortality dropping from 90.3% under *S.aureus* colonisation to 24.9% under co-colonisation with *E.faecalis* (Quasibinomial GLM. Bacterial colonisation: *F* = 192.9, df = 1, *P* < 0.01).

To investigate whether *lys-7* expression disproportionately impacts *S. aureus* colonisation over *E. faecalis* colonisation, we measured their CFUs during co-colonisation. We found a trend towards lower *S. aureus* infection loads in the wild-type host compared to in the *lys-7* knock-out mutant (Fig. 3f. *t*-test, *t* *=* 1.42, df = 8, *P* = 0.19). Although not significant, the results are consistent with our mortality data. By contrast, we found that *E. faecalis* was significantly better at colonising the wild-type host than the *lys-7* knock-out mutant (Fig. 3e. *t*-test, *t* = −3.5, df = 8, *P* = 0.0076).

## Discussion

Protective host-microbe symbioses can form naturally (Chrostek et al. 2017; Heath et al. 1999; Huigens et al. 2004; Jaenike et al. 2007), but are also artificially forged in an effort to tackle the spread of infectious disease (Bull and Turelli 2013; Magalhaes et al. 2019; Wang et al. 2017; Wilke and Marrelli 2015). In an emergent symbiosis, the host immune system may have a key role in allowing protective microbes to invade and be maintained. We investigated the effect of a novel protective microbe on the transcriptomic response of *Caenorhabditis elegans* hosts to pathogen infection. Neither the pathogen species (*S. aureus*), nor the protective microbe (*E. faecalis*), have been recorded in the native *C. elegans* microbiome, making the system valuable for studying the inception of a tripartite interaction. Under co-colonisation of protective microbe and pathogen we observed the upregulation of many nematode immune genes such as those coding for the production of antimicrobial lysozymes and C-type lectins (Dierking et al. 2016). This result reflects immune-related gene expression patterns in hosts that are naturally colonised by protective microbes (Mejía et al. 2014; Montalvo-Katz et al. 2013) as well as artificially established host-protective microbe systems (Rancès et al. 2012). The artificial symbiosis between *Wolbachia* and mosquitoes generates the upregulation of a suite of immune genes, including C-type lectins and defensin, that suppress Dengue and Chikungunya viruses (Moreira et al. 2009; Rancès et al. 2012). We also saw the upregulation of cuticular collagen genes, including *col* and *dpy* genes. Pathogens frequently produce extracellular proteins to degrade collagen in the host hypodermis (Koziel and Potempa 2013) and *C. elegans* appears to alter its cuticle structure in response (Coolon et al. 2009; Sellegounder et al. 2019; Wong et al. 2007). Upregulation of these collagen genes is essential for nematode defence against *Pseudomonas aeruginosa, Salmonella enterica* and *S. aureus* (Sellegounder et al. 2019), and has been implicated in responses to *E. faecalis, Serratia marcescens* and *Photorhabdus luminescens* (Wong et al. 2007).

Our results indicate that the nematode immune system might alter the within-host competition of *E. faecalis* and *S. aureus*, benefitting the former’s colonisation. Lysozymes are key elements of the innate immune system and catalyse the degradation of bacterial cell wall peptidoglycan and modulate the host immune response through the release of pattern recognition receptors (Ragland and Criss 2017). *E. faecalis* has been shown to be more resistant to lysozyme activity than *S. aureus* (Cisani et al. 1982), whilst lysozymes have also been found to contribute to nematode resistance against *S. aureus* (Kong et al. 2014; Visvikis et al. 2014). By knocking out *lys-7*, the most significantly upregulated lysozyme gene during co-colonisation, we found these worms had a significantly lower within-host load of protective *E. faecalis*. By contrast, there was a trend, albeit insignificant, for knock-out worms to have higher *S. aureus* colonisation. It is unclear if *lys-7* could directly benefit *E. faecalis* growth. However, a small reduction in *S. aureus* colonisation could be sufficient to indirectly impact *E. faecalis* fitness through immune-mediated competition. It was previously demonstrated that *S. aureus* drives the downregulation of *lys-7* expression in *C. elegans* and when expression is restored through chemical treatment, pathogen-induced host mortality decreased along with infection load (Kong et al. 2014). These results suggest that the upregulation of lysozyme genes in co-colonised hosts is likely to benefit *E. faecalis’* ability to colonise, and in turn, confer other mechanisms of protection, such as ROS production (King et al. 2016) and siderophore exploitation (Ford et al. 2016). Increased colonisation by *E. faecalis* has previously been shown to relate to enhanced protection in our system (King et al. 2016; Rafaluk-Mohr et al. 2018) and in other protective symbioses more generally (Drew and King 2022).

We found that the presence of *E. faecalis* was sufficient to cause the upregulation of one lysozyme and two collagen genes. Colonisation by *E. faecalis* alone caused the upregulation of the lysozyme gene, *lys-*1, and the cuticular collagen genes, *col-17* and *col-88*. Unlike this, we found that *S. aureus* alone drives the downregulation of the *lys-1* gene. Thus, *S. aureus* and *E. faecalis* appear to have opposing effects on lysozyme expression, with *E. faecalis* dominating control over *lys-1* during co-colonisation. The beneficial effect that lysozyme expression has for both *E. faecalis* and the host *C. elegans* suggests that *E. faecalis-*induced lysozyme expression could be maintained by selection in a pathogen rich environment. This mechanism of immune-mediated competition is similar to that between the commensal skin microbe, *Staphylococcus epidermis*, and harmful *S. aureus* (Pastar et al. 2020). The commensal upregulates host expression of an antimicrobial protein effective at killing the pathogen (Pastar et al. 2020), whilst *S. aureus* conversely downregulates the expression of the antimicrobial protein.

Immune upregulation caused by protective microbes might be costly to the host and thereby affect ability to spread within a host population (Oliver et al. 2008; Vorburger et al. 2013). *Wolbachia* strains introduced into novel mosquito hosts were found to upregulate host immunity, but not when in native mosquitoes. Although this upregulation enhanced protection (Rancès et al. 2012) and potentially *Wolbachia* density (Pan et al. 2018), the absence of this response in the native host suggests that its cost may outweigh its benefit (Rancès et al. 2012). When protection is inducible, rather than constitutive, it may reduce the cost to the host of maintaining the symbiont, making the symbiosis more likely to persist. In our study, we found many immune genes upregulated by *E. faecalis* in infected hosts only. These plastic changes in immune gene expression, or plastic divestment of immunity in a protective symbiosis (Martinez et al. 2016), might also reduce the cost of harbouring *E. faecalis* and prevent vulnerability to infection in future generations should the relationship break down (Ford and King 2021).

Despite the launching of host immune factors, our results suggest direct pathogen suppression from *E. faecalis* is likely still the primary means of host protection (Ford et al. 2016; King et al. 2016). Direct symbiont-pathogen interactions are common among natural protective symbioses (Ford and King 2016). Whilst *lys-7* knockout worms were highly susceptible to *S. aureus* infection experiencing 90% mortality, worms suffered less mortality during co-colonisation. This result was consistent for both knockout and wild-type worms. Thus, without *lys-7*, *E. faecalis* still confers a degree of protection, in agreement with previous findings that *E. faecalis* competes directly with *S. aureus* for iron resources via iron-binding siderophores (Ford et al. 2016) and suppresses the pathogen via superoxides (King et al. 2016). While *lys-7* was the focus of direct testing here, it is possible that other candidate genes could be contributing to protection. Further work should test other lysozyme-encoding genes (specifically *lys-2* and *lys-10*), C-type lectin and collagen-encoding genes for a strengthened test of immune-mediated competition.

Immune-mediated competition ultimately selects for the persistence of those species best able to evade the host immune factors elicited (Bjørnstad and Harvill 2005; Habets et al. 2012; Lysenko et al. 2005; Margolis et al. 2010; Raberg et al. 2006; Ulrich and Schmid-Hempel 2012). This phenomenon has been predominately examined in host-parasite systems (Raberg et al. 2006), but may be relevant for symbioses more generally. Our study shows that this process may benefit the establishment of protective microbes upon introduction to novel hosts. Specifically, we find the protective microbe was given a competitive edge from lysozyme production in a novel host species. This result may have emerged because *E. faecalis* is widely distributed among animal microbiota—as a protector (Heikkilä and Saris 2003), commensal and pathogen (Mason et al. 2011)—and lysozymes are common components of innate immune systems. Bacterial adaptations to these immune components in one host species might therefore be relevant in another. Indeed, the transfer of protective microbes to new hosts is more successful between host species that are more phylogenetically similar, i.e. their immune systems may be more similar (Russell and Moran 2005). That said, immune-mediated competition in our study could have been a convenient by-product of a triggered immune response to a novel bacterium. Our results nevertheless highlight the complexity of interactions between hosts and their microbial communities. Artificially established symbioses between animals and microbes are set to become an important tool in biocontrol (Ford and King 2016).

Identifying host factors that govern the colonisation of protective microbes will provide vital targets for approaches such as RNA interference or drug development. These developments may allow us to encourage novel symbioses to form, or indeed to disrupt existing symbioses.

## Supplementary information

Supporting_Information_Legends.pdf

SI_File1_DEGs_Saureus-vs-SaureusEfaecalis.csv

SI_File2_GOterms_Saureus-SaureusEfaecalis.csv

SI_File3_DEGs_Efaecalis-vs-OP50.csv

SI_File4_DEGs_Saureus-vs-OP50csv.csv

SI_File5_TranscriptomicReads.csv

SI_File6_StatTables.pdf

SI_File7_ImmuneFamilyDEGs.pdf

## Data Availability

Raw and processed transcriptomic data for *C. elegans* is publicly available in the Gene Expression Omnibus (GSE151731). The experimental data associated with the study is available at 10.6084/m9.figshare.c.6250053.
