# Supplementary material for: Immune-mediated competition benefits protective microbes over pathogens in a novel host species
Source: Heredity (Edinb). 2022 Nov 9;129(6):327–35. doi: 10.1038/s41437-022-00569-3 (PMC9708653; doi:10.1038/s41437-022-00569-3)
Supplement: Supplementary file 1 — Supporting_Information_Legends.pdf [file 41437_2022_569_MOESM1_ESM.pdf]

Supporting Information File Legends

SI_File1_DEGs_Saureus-vs-SaureusEfaecalis.csv
Caenorhabditis elegans genes found to be differentially regulated as a result of *Enterococcus faecalis*-mediated protection against infection by *Staphylococcus aureus* (fig. 1a). Significance in differential expression was calculated using the R package, Sleuth and defined by a q-value of <0.05 (p-value adjusted by means of the Benjamini-Hochberg false discovery date, FDR, correction for multiple comparisons). The table lists the output of a likelihood ratio test using Sleuth along with the beta values which are the effect size of differential expression.

SI_File2_GOterms_Saureus-SaureusEfaecalis.csv
The gene ontology (GO) terms that are significantly enriched in the list of *Caenorhabditis elegans* genes differentially regulated by *Enterococcus faecalis*-mediated protection. This enrichment analysis was performed using the g:Profiler online tool with the Benjamini-Hochberg FDR correction for multiple comparisons. The table lists the output of this analysis, including each gene from our dataset that is assigned to a significant GO term (listed in the column named “intersections”).

SI_File3_DEGs_Efaecalis-vs-OP50.csv
Caenorhabditis elegans genes found to be differentially regulated as a result of *Enterococcus faecalis* colonisation (fig. 1b). Significance in differential expression was calculated using the R package, Sleuth and defined by a q-value of <0.05 (p-value adjusted by means of the Benjamini-Hochberg false discovery date, FDR, correction for multiple comparisons). The table lists the output of a likelihood ratio test using Sleuth along with the beta values which are the effect size of differential expression.

SI_File4_DEGs_Saureus-vs-OP50.csv
Caenorhabditis elegans genes found to be differentially regulated as a result of *Staphylococcus aureus* infection (fig. 1c). Significance in differential expression was calculated using the R package, Sleuth and defined by a q-value of <0.05 (p-value adjusted by means of the Benjamini-Hochberg false discovery date, FDR, correction for multiple comparisons). The table lists the output of a likelihood ratio test using Sleuth along with the beta values which are the effect size of differential expression.

SI_File5_TranscriptomicReads.csv
Additional detail of transcriptomic sequencing efficiency and read assignment for all runs included in the study.

SI_File6_StatTables.pdf
Output tables from statistical analysis of host mortality and bacterial colonisation under lys-7 knockouts and wild-type N2.

SI_File7_ImmuneFamilyDEGs.pdf
Detailing of the immune gene families differentially regulated by *E. faecalis*-mediated protection in *C. elegans*
