# Supplementary material for: Immune-mediated competition benefits protective microbes over pathogens in a novel host species
Source: Heredity (Edinb). 2022 Nov 9;129(6):327–35. doi: 10.1038/s41437-022-00569-3 (PMC9708653; doi:10.1038/s41437-022-00569-3)
Supplement: Supplementary file 7 — SI_File6_StatTables.pdf [file 41437_2022_569_MOESM7_ESM.pdf]

Supplementary Information

SI Table 1. Host mortality as a function of host strain (lys-7 vs wild type N2) under *E. faecalis* colonisation

Binomial GLM	***Estimate***	***Std. Error***	***Z-value***	***P-value***	
Intercept	-4.37	0.303	-14.4	< 0. 001	
Host (N2)	-1.75	1.05	-1.67	0.094	
ANOVA chi-squared	***df***	***Dev. Res***	***df***	***Resid. Dev***	***Pr(>Chi)***
null			9	14.31	
Host	1	4.48	8	9.85	0.034

SI Table 2. Host mortality as a function of host strain (lys-7 vs wild type N2) under OP50 colonisation

Binomial GLM	***Estimate***	***Std. Error***	***Z-value***	***P-value***	
Intercept	-5.48	0.501	-10.9	< 0. 001	
Host (N2)	-19.06	5088.85	-0.004	0.997	
ANOVA chi-squared	***df***	***Dev. Res***	***df***	***Resid. Dev***	***Pr(>Chi)***
null			9	9.29	
Host	1	4.13	8	5.17	0.042

SI Table 3. Host mortality as a function of host strain (lys-7 vs wild type N2) under *S.aureus* colonisation

Quasibinomial GLM	***Estimate***		***Std. Error***	***t-value***	***P-value***	
Intercept	2.23		0.462	4.823	0.001	
Host (N2)	-1.92		0.604	-3.184	0.013	
ANOVA chi-squared	***df***	***Dev. Res***	***df***	***Resid. Dev***	***F***	***Pr(>F)***
null			9	292.4		
Host	1	167.93	8	124.5	11.2	0.0102

SI Table 4. Comparison of bacterial colonisation (CFUs) in Lys-7 knock-out host vs N2 wild-type host

F-test	***F***	***df ^{num}***	***df_{denom}***	***P -value***	***95% CI***
S.aureus	1.21	4	4	0.859	0.126-11.61
E. faecalis	7.0006	4	4	0.086	0.729 - 67.3
t-test	***t***	***df***	***P-value***	***Mean (Lys-7)***	***Mean (N2)***
S.aureus	1.42	8	0.193	86024	65037
E. faecalis	-3.54	8	0.008	14879	25294
