# Supplementary material for: Immune-mediated competition benefits protective microbes over pathogens in a novel host species
Source: Heredity (Edinb). 2022 Nov 9;129(6):327–35. doi: 10.1038/s41437-022-00569-3 (PMC9708653; doi:10.1038/s41437-022-00569-3)
Supplement: Supplementary file 8 — SI_File7_ImmuneFamilyDEGs.pdf [file 41437_2022_569_MOESM8_ESM.pdf]

Table 1. Immune gene families differentially regulated by *E. faecalis*-mediated protection in *C. elegans*. Genes are listed in order of q-value (p-values corrected by Benjamini-Hochberg FDR for multiple testing). The beta value is the effect size of differential regulation. Blue indicates downregulation whilst red indicates upregulation. Genes in bold are represented twice as unique coding sequences.

Immune gene family	Gene	Coding sequence ID	Ensemble gene	q-value	beta value
**Lysozyme**	lys-7	C02A12.4	WBGene00003096	4.71E-05	2.12E+00
	lys-2	Y22F5A.5	WBGene00003091	1.77E-03	7.84E-01
	lys-10	F17E9.11	WBGene00003099	2.25E-03	3.46E+00
	lys-3	Y22F5A.6	WBGene00003092	8.04E-03	-1.21E+00
	lys-1	Y22F5A.4.1	WBGene00003090	8.67E-03	7.68E-01
**Invertebrate lysozyme**	ilys-2	C45G7.2	WBGene00016669	6.10E-04	1.72E+00
	ilys-5	F22A3.6a	WBGene00017691	1.39E-02	8.47E-01
**C-type Lectin**	clec-264	F31D4.4	WBGene00009291	3.34E-05	-8.81E-01
	clec-5	C35D10.14	WBGene00016450	1.24E-04	8.23E-01
	clec-85	Y54G2A.6a	WBGene00021872	2.32E-04	7.97E-01
	**clec-266**	**C25B8.4a**	**WBGene00016088**	**2.53E-04**	**1.43E+00**
	clec-55	F08H9.9	WBGene00008597	2.74E-04	1.41E+00
	clec-209	Y19D10A.9	WBGene00021224	3.44E-04	1.09E+00
	clec-53	T03F1.10	WBGene00020191	3.88E-04	1.43E+00
	clec-41	B0365.6.1	WBGene00007153	3.90E-04	9.01E-01
	clec-78	F47C12.2	WBGene00018547	4.40E-04	1.81E+00
	clec-232	F36G9.11	WBGene00009487	6.30E-04	-1.21E+00
	clec-174	Y46C8AL.2	WBGene00021580	1.65E-03	2.43E+00
	clec-146	Y48E1B.9a	WBGene00013008	1.66E-03	-5.12E-01
	clec-218	W02D7.2	WBGene00020938	1.68E-03	8.02E-01
	clec-7	F10G2.3	WBGene00017364	1.70E-03	7.45E-01
	clec-82	Y54G2A.8a	WBGene00021873	2.03E-03	-6.84E-01
	clec-66	F35C5.9	WBGene00009397	2.84E-03	-4.38E-01
	clec-45	F07C4.2	WBGene00017199	3.08E-03	3.21E+00
	**clec-266**	**C25B8.4c**	**WBGene00016088**	**6.31E-03**	**1.45E+00**
	clec-50	W04E12.8	WBGene00012253	6.57E-03	6.28E-01
	clec-160	F09G8.8	WBGene00017322	7.53E-03	6.28E-01
	clec-72	Y46C8AL.5	WBGene00021583	1.17E-02	6.05E-01
	clec-180	F32E10.3b	WBGene00017991	1.36E-02	3.61E+00
	clec-196	F26D10.12a	WBGene00009156	1.49E-02	1.20E+00
	clec-210	Y73C8C.2	WBGene00022261	1.57E-02	2.48E+00
	clec-51	B0218.6a	WBGene00015050	2.12E-02	3.37E-01
	clec-1	F25B4.9	WBGene00017772	2.83E-02	6.21E-01
	clec-52	B0218.8	WBGene00015052	3.00E-02	-4.58E-01
	clec-48	C14A6.1	WBGene00007565	3.93E-02	-2.89E-01
